# Who Benefits from Acute Psychiatric Home Treatment? A Systematic Review

**DOI:** 10.1007/s10597-024-01297-0

**Published:** 2024-06-28

**Authors:** Vera Bergamaschi, Felix Baumann, Ingeborg Warnke, Salvatore Corbisiero, Fabian Ludwig, Andreas Riedel, Kerstin Gabriel-Felleiter, Stefanie J. Schmidt

**Affiliations:** 1 Institue of Psychology, University of Bern, Bern, Switzerland; 2 Luzerner Psychiatrie, Voltastrasse 42, 6005 Lucerne, Switzerland

**Keywords:** Systematic Review, Home Treatment, Predictor, Hospital Admission, Clinical Changes

## Abstract

Home treatment (HT) treats patients in an acute crisis through an interdisciplinary team with daily appointments for a short treatment period. The effectiveness of HT has already been confirmed. However, only few studies addressed specific patient characteristics associated outcome of treatment. This study aimed to identify patient characteristics associated with successful outcomes of HT. A systematic literature search was conducted according to the PRISMA guidelines. A total of 13 studies were included in the systematic review. Being employed, having a regular income, having an anxiety disorder and family involvement were associated with a successful treatment outcome in HT. High symptom severity and former hospital admissions were associated with unsuccessful treatment outcome in HT in the selected studies. HT seems to be especially beneficial for patients with paid employment or regular income, patients with anxiety disorders, and patients with familial or other social support.

## Introduction

The treatment concept of providing psychiatric care to patients in their home environment has been known for several decades (Hepp & Stulz, 2017; Hubbeling & Bertram, 2012). However, previous studies and reviews have recognized the lack of a universal definition of home-based psychiatric care (Hepp & Stulz, 2017; Sjølie et al., 2010) and provided an overview of the different approaches of home-based intervention that exist (Hepp & Stulz, 2017). One of these approaches is home treatment (HT), which targets patients in an acute psychiatric crisis who would alternatively be treated in a psychiatric hospital (Gühne et al., 2011). By definition, HT is provided by a mobile and interdisciplinary team consisting of psychiatrists, psychologists, and nurses usually with daily appointments for a short, limited treatment period. To ensure safety at home, some services provide a 24 h emergency phone (Gühne et al., 2011; Hepp & Stulz, 2017). In this study, we focus on treatment based on the aforementioned definition of HT.

Systematic literature reviews and RCTs have supported the effectiveness of HT as an alternative to conventional inpatient treatment, as the implementation of HT reduces the number of admissions to psychiatric hospitals during and after an acute psychiatric crisis (Gühne et al., 2011; Murphy et al., 2015). This was also confirmed in a recent Swiss randomised controlled trial in which over 700 adults in need of acute psychiatric treatment were randomly assigned to receive either conventional inpatient care or HT. Compared to inpatient care, HT was associated with 30% fewer days spent in hospital within two years after the initial crisis (Stulz et al., 2020).

In addition to the reduction in hospital admissions, studies focusing on changes in clinical variables found significant improvements in symptoms and level of functioning (Klug et al., 2010; Mötteli et al., 2018). These results suggest that the overall effectiveness of HT is relatively well established. However, the question remains whether HT is effective for all patients and which patient characteristics predict the effectiveness of HT.

In previous studies, severe functional impairment, comorbid disorders, younger age, unemployment, belonging to an ethnic minority, being single, and being diagnosed with a personality disorder were associated with poorer treatment outcomes of psychological treatment in an outpatient setting (Cooper & Conklin, 2015; Delgadillo et al., 2016; Lutz et al., 2021). In addition, a greater number of risk factors was significantly associated with a poorer prognosis (Lutz et al., 2021). In a review of inpatient treatments, being married, older age, and being employed significantly predicted better treatment outcomes in terms of a reduced risk of readmission. However, the number of prior inpatient treatments appeared to be a risk factor for readmission after treatment (Donisi et al., 2016).

For HT, some studies have investigated the effectiveness of HT and identified significant predictors of treatment success in secondary analyses (Barakat et al., 2021; Brimblecombe et al., 2003; Harrison et al., 2001; Kingsford & Webber, 2010). However, to the best of our knowledge, no systematic review or meta-analysis of patient characteristics that predict treatment outcome has been published. Therefore, this systematic review aims to summarize findings on patient characteristics associated with HT outcomes.

## Methods

We conducted a systematic literature search in January 2023 to identify studies that examined predictors of hospital admission after HT, readmission during HT, and changes in levels of clinical symptoms and functioning during HT. We used the "Preferred Reporting Items for Systematic Reviews and Meta-Analyses” (PRISMA) guidelines (Page et al., 2021). We searched the databases MEDLINE (PubMed), PsychINFO (Ovid), Scopus (Elsevier) and Web of Science Core Collection (Web of Science) using Medical Subject Headings (MeSH), depending on the respective database. The specific search terms are listed in the Appendix. In addition, the references of the selected studies were screened for eligibility. Two independent reviewers screened the studies for eligibility. Conflicting assessments were resolved, including the assessment of a third independent reviewer. The quality of the studies was assessed using the Cochrane ‘Risk of Bias’ assessment (see Appendix Table 2).

We included studies that focused on psychiatric HT and that performed predictor analyses for treatment outcomes in HT (hospital admission, readmission to HT, changes in symptoms and level of functioning). To ensure comparability, the treatment under investigation had to meet the above-mentioned criteria of HT: mobile, interdisciplinary treatment team, treatment in the patient’s natural environment, intensive treatment frequency (daily appointments), short treatment duration (2–6 weeks, no longer than inpatient treatment), and crisis intervention (Gühne et al., 2011; Hepp & Stulz, 2017). We included randomised controlled trials (RCTs), non-randomised trials, and intervention studies with a naturalistic design. Studies had to be published in peer-reviewed journals.

We excluded studies that investigated HT for somatic diseases and in children and adolescents. For better comparability, we did not include studies of treatment approaches related to HT, such as Assertive Community Treatment (ACT) or Community Mental Health Teams (CMHT). We also excluded reviews and book chapters.

## Results

### Study Selection

**Fig. 1:**
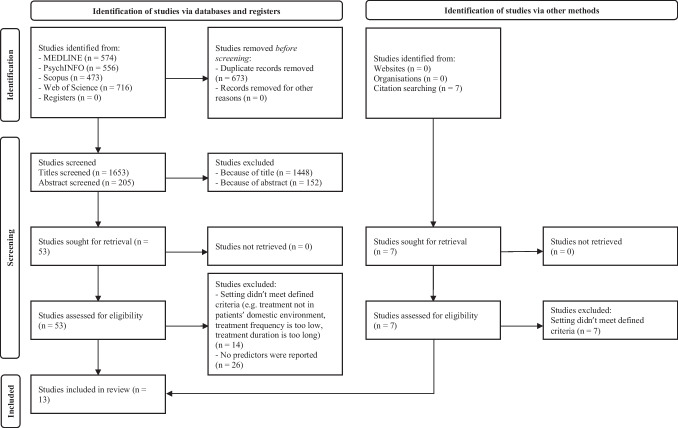
Flow chart of the systematic literature search

The systematic literature search yielded 574 studies for PubMed, 556 for PsycINFO, 473 for Scopus and 716 for Web of Science. The initial search therefore yielded a total of 2326 studies, of which 7 studies were added by cross-referencing. After removing duplicates, a total of 1653 studies remained (see Fig. 1). The titles of the studies were screened first. The abstracts of the remaining 205 studies were screened, resulting in the exclusion of a further 152 studies. After reviewing the full texts of the remaining 53 studies, 40 studies were excluded because they either did not meet the defined criteria for HT or did not report predictors of treatment success in HT. 13 studies were included in the systematic review (see Table 1).

**Table 1:** Overview of all studies included

Authors (year)	Study Origin	Design	Sample size (n), age, percentage female	Measures of predictors	Outcome	Summary of results
Biong et al. (2012)	Norway	Observational study)	n = 363 Mean age: a majority (72%) in the age group of 26 to 65 years Female: 65%	Symptom severity: Health of the Nation Outcome Scale (HoNOS) (Wing et al., 1998, 1999) Level of functioning: Global Assessment of Functioning Scales (GAF) both for symptoms (GAF-S) and functioning (GAF-F) (Endicott et al., 1976; Goldman et al., 1992; Karterud et al., 1998)	A) Changes in morbidity and clinical problems during HT B) Chances in HoNOS and GAF	Income source was significantly associated with more changes in symptoms and functioning (outcome B): GAF-S changes are significantly different (F = 5.56, *p* = 0.004) between regular income group and sick/disability pay group
						Age, gender, marital status, and living situation had no significant effect on outcomes A and B
Brimblecombe et al. (2003)	UK	Observational study	n = 293 Mean age (range): 42.2 years (17–88) Female: 67%	Extent of psychiatric impairment: Brief Psychiatric Rating Scale (BPRS) (Overall & Gorham, 1962) Suicide ideation: Scale for Suicide Ideation (SSI) (Beck et al., 1979)	Hospital admission during HT	High suicidal ideation was associated with more hospital admissions during HT (*p* < 0.01) Previous hospital admissions were associated with more hospital admissions during HT (*p* < 0.01)
						Diagnosis, gender, living situation and employment status had no significant effect on outcome
Córcoles et al. (2015)	Spain	Non-randomized trial	n = 896 Mean age SD: 45.6 years (17.9) Female: 42%	Illness severity: Severity of Psychiatric Illness scale (SPI) (Bulbena et al., 1997) Level of functioning: Global Assessment of Functioning Scale (GAF) (Hall, 1995)	Hospital admission within 1y after HT	Family involvement was associated with fewer hospital admissions within 1y after HT (*OR* = 0.53, 95% CI [0.32, 0.89], *p* = 0.015) High symptom severity was associated with more hospital admissions within 1y after HT (*OR* = 12.10, 95% CI [4.83, 30.28), *p* < 0.001)
Hasselberg et al. (2013)	Norway	Non-randomized trial	n = 680 Mean age (SD): 41.8 years (14.7, patients with hospital admission) / 39.6 years (15.1, patients with no hospital admission) Female: 58% (patients with hospital admission) / 59% (patients with no hospital admission)	Symptom severity: Health of the Nation Outcome Scale (HoNOS) (Wing et al., 1998) Level of functioning: Global Assessment of Functioning Scale (GAF) (Goldman et al., 1992)	Hospital admission during HT	Psychotic symptoms were associated with more hospital admissions during HT (*OR* = 16.46, 95% CI [4.97, 54.55], *p* = *0.*001) Severe to very severe depressive symptoms were associated with more hospital admissions during HT (*OR* = 2.77, 95% CI [1.17, 6.57], *p* = 0.021) Suicide risk was associated with more hospital admissions during HT (*OR* = 3.75, 95% CI [2.21, 6.33], *p* < 0.001) Previous hospital admissions within the last 12 month before HT were associated with more hospital admissions during HT (*OR* = 3.85, 95% CI [2.42, 6.13]* p* < 0.001)
						Age, gender, living alone, substance misuse and risk to others had no significant effect on outcome
Huang et al. (2018)	UK	Observational Study	n = 309 Mean age (SD): 40.4 years (12.0) Female: 49%	Symptom severity, improvement, and treatment response: Clinical Global Impression (CGI) Scale (Guy, 2000)	A) Changes in symptom severity during HT B) Hospital admission during HT	High symptom severity at beginning of treatment was associated with more hospital admissions during HT (outcome B) (*p* < 0.05) No medications or non-concordance with medication were associated with more hospital admissions during HT (outcome B) (*χ*^*2*^ = 43.3) compared with one medication
						Age, gender, number of medications prescribed, dru, and alcohol status had no significant effect on outcome A
Kingsford and Webber (2010)	UK	Observational study	n = 260 Mean age (SD): 41.4 years (12.8) Female: 55%	Social deprivation: Index of multiple deprivation (IMD) (Office of the Deputy Prime Minister, 2004)	Hospital admission during HT	Higher age was associated with more hospital admissions during HT (*p* = 0.02) Living in more socially deprived areas was associated with more hospital admissions during HT (*p* = 0.04)
						Gender, ethnicity and living situation had no significant effect on outcome
Leon-Caballero et al. (2020)	Spain	Observational study	n = 304 Mean age (SD): 45.1 years (16.9) Female: 53%	Symptom severity, improvement, and treatment response: Clinical Global Impression (CGI) Scale* Level of functioning: Global Assessment of Functioning Scale (GAF)* Psychotic symptoms: Positive and Negative Syndrome Scale (PANSS)* Manic symptoms: Young Mania Rating Scale (YMRS)* Depressive Symptoms: Hamilton Depression Rating Scale (HDRS)*	Readmission to HT or impatient setting within 90 days after HT	High symptom severity was associated with more readmissions to HT or inpatient setting within 90 days after HT (statically significant but no numbers presented) High level of functionality was associated with less readmissions to HT or impatient setting within 90 days after HT (statically significant but no numbers presented) Suicidal behaviour was associated with more readmissions to HT or impatient setting within 90 days after HT (statically significant but no numbers presented) Manic symptoms were associated with more readmissions to HT or impatient setting within 90 days after HT (statically significant but no numbers presented)
Ma et al. (2021)	UK	Observational study	n = 224 Mean age (SD): 40.0 years (12.5) Female: 60%	Loneliness: University of California at Los Angeles Loneliness Scale (ULS-8) (Hays & DiMatteo, 1987) Social isolation: Lubben Social Network Scale (LSNS-6) (Lubben et al., 2006) Recovery process: Questionnaire about the process of recovery (QPR) (Law et al., 2014; Neil et al., 2009; Williams et al., 2015) Extent of psychiatric impairment: Brief Psychiatric Rating Scale (BPRS) (Overall & Gorham, 1962)	Self-rated personal recovery during HT	Persistent severe loneliness was associated with less recovery during HT (*b* = -12.83, 95% CI [-18.83, -6.83], *p* < 0.001) Long duration of illness (2–10 years since first contact with mental health services) was associated with less recovery during HT (*b* = -8.2, 95% CI [-13.49, -2.91], *p* = 0.003)
						Social network size, age, gender, ethnicity, educational attainment, number of psychiatric impatient admissions and first contact with mental health service within 2 years had no significant effect on outcome
Mötteli et al. (2018)	Switzerland	Observational study	n = 201 (HT group) Mean age (SD): 44.4 years (12.1) Female: 66%	Symptom severity: Health of the Nation Outcome Scale (HoNOS) (Wing et al., 1998) Level of functioning: Global Assessment of Functioning Scale (GAF) (Jones et al., 1995) Symptom severity, improvement, and treatment response: Clinical Global Impression (CGI) Scale (Busner & Targum, 2007)	Hospital admission during or after HT	Substance use disorder (F1) was associated with more hospital admissions during or after HT (*p* < 0.05) Affective disorder (F3) was associated with fewer hospital admissions during or after HT (*p* < 0.05)
Mötteli et al. (2020)	Switzerland	Observational study	n = 408 (n = 186 site 1 / n = 222 site 2) Mean age (SD): 44.5 years (12.3, site 1) / 41.6 years (12.7, site 2) Female: 67% (site 1) / 69% (site 2)	Symptom severity: Health of the Nation Outcome Scale (HoNOS) (Wing et al., 1998)	A) Hospital admission during HT B) Changes in symptom severity during HT C) Successful replacement of hospital care (> 50% of total treatment episode spent in HT, HT duration < 40d, agreement on HT termination was mutual)	Employment was associated with more successful replacements of hospital care (outcome C) (Site 1: *OR* = 2.40, 95% CI [1.16, 4.94], *b* = 0.87, *p* < 0.05) / (Site 2: *OR* = 2.05, 95% CI [1.14, 3.68], *b* = 0.72, *p* < 0.05) High HoNOS scores at baseline were associated with less successful replacements of hospital care (outcome C) (Site 1: *OR* = 0.94, 95% CI [0.89, 1.00], *b* = -0.06, *p* < 0.05) / (Site 2: *OR* = 0.95, 95% CI [0.90, 1.00], *b* = -0.06, *p* < 0.05) Direct admission to HT was associated with more successful replacements of hospital care (outcome C) (Site 1: *OR* = 0.37, 95% CI [0.19, 0.75], *b* = -0.98, *p* < 0.01) / (Site 2: *OR* = 0.12, 95% CI [0.05, 0.32], *b* = -2.09, *p* < 0.001)
						Female gender, older age, living together with others and affective disorder as main psychiatric disorder had no significant effect on outcome C
Munz et al. (2011)	Germany	Non-randomized trial	n = 60 (HT Group) Mean age (SD): 39.8 years (14.0) Female: 70%	Psychotic symptoms: Positive and Negative Syndrome Scale (PANSS) (Kay et al., 1989) Depressive symptoms: Hamilton Depression Rating Scale (HAMD-21) (Hamilton, 1960) Symptom severity; Health of the Nation Outcome Scale (HoNOS) (Andreas et al., 2007)	A) Improvement in psychotic symptoms B) Changes in level of functioning during HT	Increased psychotic symptoms at baseline were associated with better improvement in psychotic symptoms (outcome A) compared to fewer psychotic symptoms at baseline (*b* = -.32, *p* = 0.000) Increased functional impairments at baseline were associated with less improvement in psychotic symptoms (outcome B) compared to little symptom severity at baseline (*b* = 0.29, *p* = 0.003)
Turhan and Taylor (2016)	UK	Observational study	n = 64 (only patients with borderline PS disorder) Mean age (SD): 40.8 years (no value reported) Female: 100%	Symptom severity, improvement, and treatment response: Clinical Global Impression (CGI) Scale (Busner & Targum, 2007)	Hospital admission during HT	Improvement in symptom severity was associated with fewer hospital admissions during HT (*p* < 0.000)
Werbeloff et al. (2017)	UK	Observational study	n = 17666 Mean age: a majority (69% both sites each) in the age group of 25 to 54 years Female: 52% (site 1) / 55% (site 2)	Symptom severity: Health of the Nation Outcome Scale (HoNOS) (Wing et al., 1998)	Hospital admission within 1y after HT	Older age (65y +) was associated with more hospital admissions within 1y after HT ↑ (Site 1: *HR* = 1.18, 95% CI [1.01, 1.37]) / (Site 2: *HR* = 1.32, 95% CI [1.12, 1.56]) First contact with HT was associated with fewer hospital admissions within 1y after HT (Site 1: *HR* = 0.57, 95% CI [0.52, 0.62]) / (Site 2: *HR* = 0.69, 95% CI [0.63, 0.75]) Diagnosis of a psychotic disorder was associated with more hospital admissions within 1y after HT (Site 1: *HR* = 1.25, 95% CI [1.09, 1.44]) / (Site 2: *HR* = 1.27, 95% CI [1.17, 1.38]) Diagnosis of an anxiety disorder was associated with fewer hospital admissions within 1y after HT (Site 1: *HR* = 0.81, 95% CI [0.69, 0.96]) / (Site 2: *HR* = 0.77, 95% CI [0.67, 0.87])
						Social deprivation had no significant effect on outcome

*HT* home treatment

^*^ no citation available

### Main Results

The following section presents the results of the 13 selected studies (see Table 1). The studies were published between 2003 and 2021, with all but one having been published since 2010. Half of the studies (n = 6) were conducted in the United Kingdom, two studies each in Norway, Spain, and Switzerland, and one study in Germany. Different study designs were included: non-randomized studies (n = 3) and observational studies (n = 10), including two cohort studies.

Regarding the study population, only 10 studies reported the mean age of their participants (M = 42.2 years). Two studies categorised patients into age groups rather than reporting their exact age. They found that the majority of participants were between 26 and 65 years old (72%) and between 25 and 54 years old (69%) (Biong et al., 2012; Werbeloff et al., 2017). On average, 62% of participants were female, although one study had an all-female population (Turhan & Taylor, 2016).

A total of 13 different measurement tools were used to assess treatment outcomes (see Table 1). Nine studies defined hospital admission as the primary outcome for HT. One study also reported successful replacement of hospital care. Treatment was defined as successful if more than 50% of the total treatment episode was spent in HT, the treatment duration was less than 40 days, and the agreement on HT termination was mutual between the patient and the team. Changes in clinical variables (symptom severity, clinical problems, level of functioning, self-rated personal recovery) were reported as outcomes in five studies.

#### Sociodemographic Variables

The majority of studies (n = 7) reported nonsignificant results for all sociodemographic variables. Significant associations with treatment outcome were reported only for age, source of income or employment, and place of residence. Two studies found that older age was significantly associated with more hospital admissions during HT and within one year after HT (Kingsford & Webber, 2010; Werbeloff et al., 2017). However, several studies reported no significant effect between age and treatment outcomes (Biong et al., 2012; Hasselberg et al., 2013; Huang et al., 2018; Ma et al., 2021; Mötteli et al., 2020).

In addition, two studies found a positive association between income source and treatment outcome: employed patients with a regular income had a better level of functioning at discharge and therefore a better recovery compared to patients on sick leave and those on disability pay (Biong et al., 2012). Employment was also significantly correlated with successful hospital replacement by HT (Mötteli et al., 2020). Employed patients were more likely to spend more than 50% of their treatment time in HT, to have a treatment duration of less than 40 days, and to end HT by mutual agreement with the treatment team (Mötteli et al., 2020). However, one study also found an insignificant association between employment status and hospital admission (Brimblecombe et al., 2003).

Regarding place of residence, living in more socially deprived areas was significantly associated with more hospital admissions (Kingsford & Webber, 2010). No significant associations were reported for gender, ethnicity, marital status, living arrangements (living alone or with others), and educational attainment (Biong et al., 2012; Brimblecombe et al., 2003; Hasselberg et al., 2013; Huang et al., 2018; Kingsford & Webber, 2010; Ma et al., 2021; Mötteli et al., 2020).

#### Diagnosis and Psychopathology

Significant associations were found for the following clinical symptoms and diagnoses: psychotic, manic, anxious, and depressive symptoms, and substance use. In particular, two studies reported a significant positive association between psychotic symptoms and hospital admissions during HT and within one year of discharge from HT (Hasselberg et al., 2013; Werbeloff et al., 2017). In contrast, one study reported that patients with more psychotic symptoms at baseline had greater reductions in psychotic symptoms during HT than patients with fewer psychotic symptoms (Munz et al., 2011).

One study found a positive association between manic symptoms and readmission to hospital within 90 days after HT (Leon-Caballero et al., 2020). For anxiety, meeting the diagnostic criteria for an anxiety disorder as a primary diagnosis was significantly associated with a reduced risk of hospital admission within one year of discharge from HT (Werbeloff et al., 2017). Mixed results were reported for depressive symptoms. Patients with severe depressive symptoms were more likely to be admitted to hospital during HT than patients with mild depressive symptoms (Hasselberg et al., 2013). On the other hand, the diagnosis of an affective disorder was not significantly associated with successful replacement of hospital care by HT (Mötteli et al., 2020). Furthermore, patients who were hospitalized during HT were even less likely to have an affective disorder than patients who were not hospitalized (Mötteli et al., 2018).

In one study, substance use correlated positively with hospital admission during and after HT. Patients admitted to hospital were more likely to have a substance use disorder than those not admitted (Mötteli et al., 2018). In addition, two studies found nonsignificant results for substance use for hospital admission during HT (Hasselberg et al., 2013) and for changes in symptom severity (Huang et al., 2018).

One older study found no significant difference in hospital addmission rates between diagnoses in general (Brimblecombe et al., 2003). Regardless of diagnosis, three studies reported that severely ill patients were more likely to be admitted to hospital during HT than less ill patients (Córcoles et al., 2015; Huang et al., 2018; Leon-Caballero et al., 2020). In addition, patients with high symptom severity over time were more likely to be admitted to hospital than those with symptom improvement during HT (Turhan & Taylor, 2016).

#### Level of Functioning

Three studies reported significant correlations between the level of functioning at the start of HT and treatment outcome: A low level of functioning at baseline was significantly associated with a high risk of readmission to HT or inpatient setting within 90 days after discharge from HT (Leon-Caballero et al., 2020). In addition, patients with a higher level of functioning at baseline had a more successful outcome of HT, defined by fewer hospital days during HT, shorter treatment duration, and more frequent withdrawal by mutual agreement than patients with a lower level of functioning (Mötteli et al., 2020). Consistent with this, patients with a low level of functioning at the start of HT showed less improvement in their psychotic symptoms over time than patients with a higher level of functioning (Munz et al., 2011).

#### Suicidality and Risk to Others

Two studies found a significant positive association between higher suicidal ideation and more hospital admissions during HT (Brimblecombe et al., 2003; Hasselberg et al., 2013). One study showed an increased risk of readmission to HT within 90 days of discharge for patients with suicidal behaviour (Leon-Caballero et al., 2020). However, risk to others was not significantly associated with hospital admission during HT (Hasselberg et al., 2013).

#### Medication Use

Patients’ medication use during HT in relation to HT outcomes was reported in only one study (Huang et al., 2018). They reported a significant positive association between both medication non-compliance and no medication use and hospital admission during HT. The authors did not specify the type of medication for these results. In the same study, the number of medications used was not a significant predictor of improvement in symptom severity.

#### Previous Hospital Admission

Previous hospital admissions and previous inpatient care also had an impact on treatment outcome. Two studies found that previous hospital admissions were significantly correlated with more hospital admissions during current HT (Brimblecombe et al., 2003; Hasselberg et al., 2013). Consistent with these findings, first contact with HT services was significantly correlated with lower rates of hospital admission within one year of discharge from HT (Werbeloff et al., 2017). Patients admitted directly to HT were also significantly more likely to have successful replacements of hospital care than patients who were transferred from inpatient care to HT (Mötteli et al., 2020). In addition, patients with a long psychiatric history had a poorer recovery than those with a short or no psychiatric history. Patients whose first contact with mental health services was between two and ten years ago showed less self-rated recovery at 18 months after HT than patients whose first contact was less than three months ago (Ma et al., 2021). In the same study, both number of psychiatric inpatient admissions and first contact with mental health services within the previous two years were found to have non-significant effects on self-rated recovery at 18-month follow-up.

#### Family Involvement

Family involvement was inconsistently associated with both outcome variables, hospital admissions and changes in clinical variables. In one study, family involvement was significantly negatively correlated with hospital admission within one year of HT discharge (Córcoles et al., 2015). Thus, socially embedded patients were less likely to be hospitalised than those without family involvement. Social deprivation was also mentioned as an insignificant predictor of hospital admissions within one year of HT discharge (Werbeloff et al., 2017). In addition, loneliness predicted less successful HT. Patients with perceived persistent loneliness rated their recovery worse at 18 month follow-up than patients who felt socially embedded (Ma et al., 2021). In the same study, the size of the social network was not significantly associated with self-rated recovery.

### Risk of Bias

Study quality was assessed using the Cochrane ‘Risk of Bias Assessment’ (Higgins et al., 2019), see Appendix 1. Selection bias was assessed for observational and non-randomized studies. Performance bias and detection bias were not assessed as blinding is not possible in this area of research. All included studies were at risk of selection bias. One study showed signs of attrition bias due to incomplete outcome data (Ma et al., 2021) and two studies showed reporting bias due to selective reporting of outcomes (Córcoles et al., 2015; Turhan & Taylor, 2016).

## Discussion

The aim of this systematic review was to summarize studies that describe patient characteristics that are associated with the outcome of HT. Treatment outcome was defined as hospital admission during and after HT, readmission to HT, change in clinical variables or change in level of functioning during HT.

We included 13 studies that reported sociodemographic variables, diagnoses and psychopathology, suicidality, risk to others, symptom severity, medication use, previous hospital admissions, functional level at baseline, and social embeddedness in addition to their evidence of the effectiveness of HT. We examined their association with successful HT outcomes. The following variables significantly predicted unsuccessful HT outcomes: older age, more severe symptoms, and previous hospital admissions. In addition, people living in socially deprived areas, having more psychotic, manic and major depressive symptoms, substance misuse, suicidality, non-adherence or non-compliance with medication, low level of functioning at baseline, and persistent loneliness were found to be at risk of unsuccessful HT outcomes in this review. In contrast, employment, anxiety disorder, and positive family involvement predicted successful HT.

### Employment

Our study results indicate that employed patients with a normal source of income had more successful outcomes of HT than patients who were on sick leave or receiving disability pay. In addition, in line with a study of intensive HT provision, income level was relevant to treatment outcome. Patients with high income were significantly less likely to be admitted to hospital than those with low income (Barakat et al., 2021). Income level a priori determines access to health care according to a current review (Tzenios, 2019). In particular, low income is one of the main obstacles to accessing of healthcare, which could lead to further deterioration in health status. In line with this, recent research during the COVID-19 lockdown showed worse mental health status and more perceived stress for people without work during the lockdown period (Pieh et al., 2020).

### Family Involvement

Family involvement was a factor that positively influenced treatment outcome in HT. Our review showed that patients whose families were involved were less often hospitalised than those who were socially isolated. For patients with severe mental illness, family involvement appeared to be beneficial, as families of severely ill patients could provide moral and practical support and motivation for recovery (Aldersey & Whitley, 2015). In addition, the patient’s social environment could identify potential crises at an early stage, which may reduce the likelihood of a relapse (Barakat et al., 2021). However, a recent intervention study of a crisis management and HT team found no significant difference in treatment outcomes despite family involvement during treatment (van Oenen et al., 2018).

### Psychopathology

A diagnosis of an anxiety disorder was associated with a better outcome of HT. Outpatient cognitive behavioral therapy (CBT) for anxiety disorders was reported to be highly effective in meta-analysis (Hans & Hiller, 2013). In vivo exposure as part of CBT has well documented success in the treatment of anxiety disorders (Kaczkurkin & Foa, 2015). HT, as a treatment in the patient’s domestic environment, allows for the integration of exposure exercises into everyday life under real-life conditions (Hepp & Stulz, 2017), which may explain the success of HT in patients with anxiety disorders.

However, symptom severity was a significant risk factor for unsuccessful treatment outcome, which was supported by many of the studies in this review. Patients with more severe symptoms were more likely to be admitted to hospital than those with mild or no symptoms. And severe symptoms such as suicide risk, suicide plans, and non-suicidal self-harm were also significantly associated with admissions to hospital during HT. Patients whose symptoms improved during treatment were less often hospitalised than those whose symptoms did not improve. Consistent with our findings, severe symptoms such as overactivity, aggressiveness, disruptiveness, and agitation were positively correlated with admission in a study of intensive HT. Patients with these symptoms, some of which were dangerous to others, had a 36% increased risk of being admitted to hospital during HT compared with patients without such symptoms (Barakat et al., 2021).

### Psychiatric History

Another factor associated with unsuccessful treatment outcomes was previous hospital admissions. Patients with previous hospital admissions and a long history of psychiatric hospitalisation were more likely to have new and increased hospital admissions during and after the current HT. They were also less often successfully discharged from HT. These findings are consistent with those from the inpatient setting (Donisi et al., 2016). In HT, patients who had previously been involuntarily hospitalized were very likely to be readmitted to inpatient care within six weeks of the onset of their crisis. Furthermore, comparing patients who were and were not admitted to hospital during treatment showed that patients in the non-admitted group had more visits at the General Practitioner in the year prior to HT (Barakat et al., 2021).

Some of the predictors presented, such as limited family involvement, higher symptom severity, and longer hospital stays, are not limited to HT, but are also known to be predictors of poorer treatment outcomes in inpatient and outpatient settings. As these predictors occurred across different settings and different disorders, they may rather function as indicators of overall global severity of illness (Zimmerman et al., 2018). As reported in this review, the overall severity of illness had a major impact on treatment outcome: higher risk of readmission and less improvement in disorder-specific symptoms (Leon-Caballero et al., 2020; Munz et al., 2011). It is therefore important that patients showing these unfavourable predictors of treatment outcome receive appropriate help and support.

### Strengths and Limitations

#### Strengths

This is the first systematic review to summarise the findings on patient characteristics associated with the outcome of HT. The definition of treatment outcome distinguishes between hospital admission, changes in clinical variables, and changes in level of functioning. This allows differential conclusions about the significance of the predictors of outcome analysed in HT.

#### Limitations

Although HT is a widely used treatment approach, variations in its implementation across countries and providers may limit the generalisability of study results. To facilitate comparability, inclusion and exclusion criteria for this literature review were defined according to current research on HT (Gühne et al., 2011; Hepp & Stulz, 2017). Nevertheless, the selected treatment approaches showed slight variations in team composition, treatment intensity, and treatment duration. For example, in one study examining different HT services in Norway, only four out of eight crisis resolution teams offered daily appointments. In the other four HT services, treatment teams only worked during office hours (Hasselberg et al., 2013). In addition, not all services provided a 24-h emergency number, which further contributed to the heterogeneity of the treatment modalities assessed. An additional challenge was the use of different names and labels for treatment services. The search term was defined as broadly as possible to include all relevant treatment services. Nevertheless, some studies that examined a comparable setting may have been missed due to differences in terminology. The lack of a common definition of effectiveness in the selected studies underlines the need for careful interpretation of the results. The definition of effectiveness varied widely, ranging from comparisons of inpatient settings on routine clinical variables to changes in clinical variables assessed with questionnaires.

The studies included in this review generally had heterogeneous samples, focusing on patients across the whole psychiatric spectrum. However, one study only focused on patients with a borderline personality disorder, resulting in an overrepresentation of this patient group in the results section (Turhan & Taylor, 2016). Furthermore, the two studies by Mötteli et al., (2018, 2020) partially used the same sample. Both studies used patient data from the HT service in Switzerland with different research questions. As a result, the sample size as well as the gender ratio included in each study differed slightly.

In terms of methods, some studies did not report whether the results reached a level of significance. In addition, the risk of bias assessment revealed possible biases in some of the selected studies, which limits the interpretation of the results. In particular, selection bias could reduce the generalizability because it is possible that only patients who were considered clinically suitable candidates for HT were included. This selection bias is known to be especially pronounced in observational studies and non-randomized trials (Grimes & Schulz, 2002), which was the design of all of the studies selected for this review. Therefore, studies with quasi-randomized or randomized controlled designs are needed.

Finally, except for symptom severity and previous hospital admissions, most of the identified predictors could only be supported by single significant results. Thus, the small number of studies and the fact that the selected predictor analyses were secondary analyses are further limitations.

### Future research

To deepen the understanding of the predictors discussed in this review, future research could focus on whether the predictors found are based on a common factor that is considered to be characteristic of an overall severity of illness. In addition, future research should examine the association of the predictors with changes in clinical variables during HT, as previous studies have manly examined whether (re-)admissions to hospital occur. Although the success of HT is well documented, future research could focus on the underlying factors to further understand the reasons for the success of this form of treatment. It would be interesting to understand the mechanisms related to the therapeutic alliance, as patients are often seen by several members of the interdisciplinary team. Overall, future research should focus on quasi- or randomized controlled study designs to enhance generalizability of the effects and predictors found in this review.

### Conclusion

More than 40 years after its introduction, the successful implementation of HT has been widely documented. Recent research suggests that certain patient variables, including employment, certain diagnoses such as anxiety disorders, symptom severity, previous hospital admissions, and family involvement, may predict the treatment success in HT. Identifying these predictors will enable healthcare professionals to develop appropriate intervention strategies to reduce hospital admissions and improve clinical outcomes by clarifying the specific treatment needs of each patient group.

## Figures and Tables

**Table:**
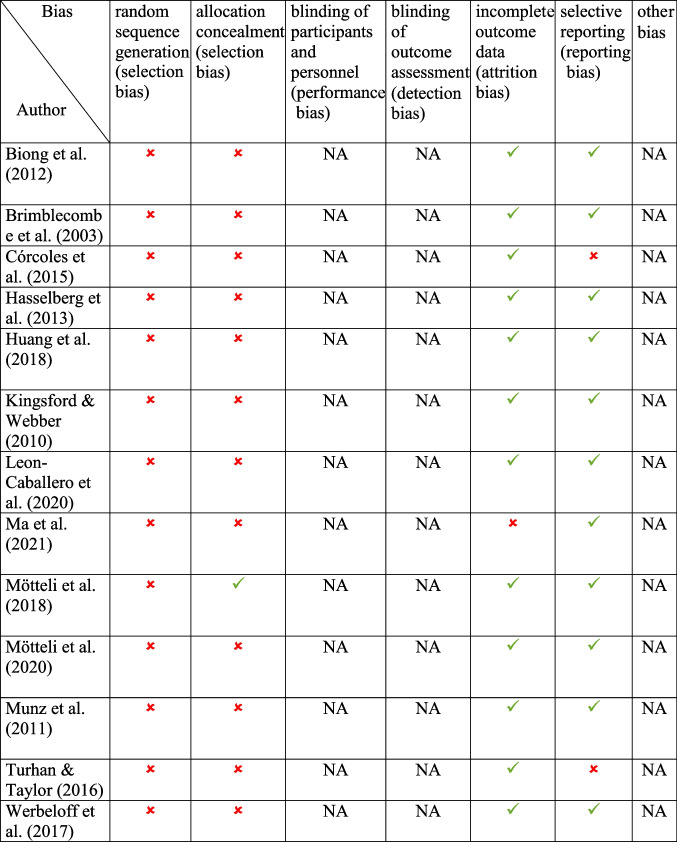
Cochrane ‘risk of bias’ assessment

Bias not present or bias removed,  Bias present, *NA* not applicable

## Appendix 1

Table 2
.

## Appendix 2. Specific search terms sorted by database:

- MEDLINE (PubMed): ("hometreatment" OR "home treatment" OR "crisis resolution") AND ("mental disorders" [mesh] OR mental health* OR psych* OR crisis) NOT (adolescent [mesh] OR child [mesh])
- PsycINFO (Ovid): (((home treatment or hometreatment or crisis resolution) and (crisis or mental disorder* or mental health* or psych*)) not (child* or adolesc*)).mp. [mp = title, abstract, heading word, table of contents, key concepts, original title, tests & measures, mesh]
- Scopus (Elsevier): TITLE-ABS-KEY(("home treatment" OR Hometreatment OR "crisis resolution") AND (crisis OR mental AND disorder* OR mental AND health OR psych*) AND NOT (child* OR adolesc*)
- Web of Science Core Collection (Web of Science): (("hometreatment" OR "home treatment" OR "crisis resolution") AND (mental disorder* OR mental health* OR psych* OR crisis) NOT (adolesc* OR child*))
